# Temporal and geographic evidence for evolution of Sin Nombre virus using molecular analyses of viral RNA from Colorado, New Mexico and Montana

**DOI:** 10.1186/1743-422X-6-102

**Published:** 2009-07-14

**Authors:** William C Black, Jeffrey B Doty, Mark T Hughes, Barry J Beaty, Charles H Calisher

**Affiliations:** 1 Department of Microbiology, Immunology & Pathology, College of veterinary Medicine and Biomedical Sciences, Colorado State University, Fort Collins, Colorado, USA; 2 Arthropod-borne and Infectious Diseases Laboratory, Department of Microbiology, Immunology & Pathology, College of veterinary Medicine and Biomedical Sciences, Colorado State University, Fort Collins, Colorado, USA

## Abstract

**Background:**

All viruses in the family *Bunyaviridae *possess a tripartite genome, consisting of a small, a medium, and a large RNA segment. Bunyaviruses therefore possess considerable evolutionary potential, attributable to both intramolecular changes and to genome segment reassortment. Hantaviruses (family *Bunyaviridae*, genus *Hantavirus*) are known to cause human hemorrhagic fever with renal syndrome or hantavirus pulmonary syndrome. The primary reservoir host of Sin Nombre virus is the deer mouse (*Peromyscus maniculatus*), which is widely distributed in North America. We investigated the prevalence of intramolecular changes and of genomic reassortment among Sin Nombre viruses detected in deer mice in three western states.

**Methods:**

Portions of the Sin Nombre virus small (S) and medium (M) RNA segments were amplified by RT-PCR from kidney, lung, liver and spleen of seropositive peromyscine rodents, principally deer mice, collected in Colorado, New Mexico and Montana from 1995 to 2007. Both a 142 nucleotide (nt) amplicon of the M segment, encoding a portion of the G2 transmembrane glycoprotein, and a 751 nt amplicon of the S segment, encoding part of the nucleocapsid protein, were cloned and sequenced from 19 deer mice and from one brush mouse (*P. boylii*), S RNA but not M RNA from one deer mouse, and M RNA but not S RNA from another deer mouse.

**Results:**

Two of 20 viruses were found to be reassortants. Within virus sequences from different rodents, the average rate of synonymous substitutions among all pair-wise comparisons (π_s_) was 0.378 in the M segment and 0.312 in the S segment sequences. The replacement substitution rate (π_a_) was 7.0 × 10^-4 ^in the M segment and 17.3 × 10^-4 ^in the S segment sequences. The low π_a _relative to π_s _suggests strong purifying selection and this was confirmed by a Fu and Li analysis. The absolute rate of molecular evolution of the M segment was 6.76 × 10^-3 ^substitutions/site/year. The absolute age of the M segment tree was estimated to be 37 years. In the S segment the rate of molecular evolution was 1.93 × 10^-3 ^substitutions/site/year and the absolute age of the tree was 106 years. Assuming that mice were infected with a single Sin Nombre virus genotype, phylogenetic analyses revealed that 10% (2/20) of viruses were reassortants, similar to the 14% (6/43) found in a previous report.

**Conclusion:**

Age estimates from both segments suggest that Sin Nombre virus has evolved within the past 37–106 years. The rates of evolutionary changes reported here suggest that Sin Nombre virus M and S segment reassortment occurs frequently in nature.

## Background

When Sin Nombre virus (SNV; family *Bunyaviridae*, genus *Hantavirus*), the causative agent of the then newly recognized hantavirus pulmonary syndrome in humans, was discovered in 1993 in New Mexico, Colorado, and Arizona, the next step in understanding the links in the chain of transmission was to determine its natural history [1]. All other hantaviruses recognized to that time had been shown to be associated with wild rodents and therefore efforts were focused on rodents. It was soon shown that the deer mouse, *Peromyscus maniculatus*, is the reservoir host of this virus [2] and has since been shown that each hantavirus is associated with rodents or insectivores of single or a scant few species in long-term, perhaps co-evolutionary, relationships [3].

Subsequent investigations of genotypes of North American hantaviruses, principally of SNV, have indicated or suggested that, virus lineages occur in relative, if discontinuous geographic isolation and may yet be monophyletic, irrespective of geographic distribution. This has been attributed to rodent host genetics [3]. In addition, viral phylogeographic differences may be correlated with deer mouse phylogeographic differences [4] and a variety of complex interactions may lead to genetic diversity of both the rodent hosts and the viruses [5].

As with all viruses assigned to the *Bunyaviridae*, hantaviral genomes comprise three RNA segments: a large (L) RNA, a medium (M) RNA, and a small (S) RNA. The L RNA encodes the polymerase gene, the M RNA encodes a precursor polyprotein for the two virion glycoproteins Gn and Gc and a nonstructural protein NSm, and the S RNA encodes the nucleocapsid protein. Dual infections of cells with closely related hantaviruses can yield reassortant viruses (a mixture of RNA genome segments of the two viruses) and reassortant viruses have potential epidemiologic implications [6,7].

Reassortants of SNV have been identified from field-collected deer mice and from dually infected cells in vitro [8-10]. The authors of those reports suggested that reassortment with heterologous hantaviruses does not occur at all or is rare but that segment reassortment in SNV-infected deer mice might occur fairly regularly.

Such complexities and opportunities suggested to us that it would be of value to analyze the RNAs of SNV from deer mice in areas of select western U.S. states (Colorado, New Mexico and Montana) characterized by similar and different habitat types. We expected that the results of such evaluations would provide insight to the geographic distribution, movement, and evolution of this virus. The studies reported here demonstrate that SNV reassortment occurs frequently and that it occurs at a high rate for both the small and medium RNA segments.

## Results

**Figure 1:**
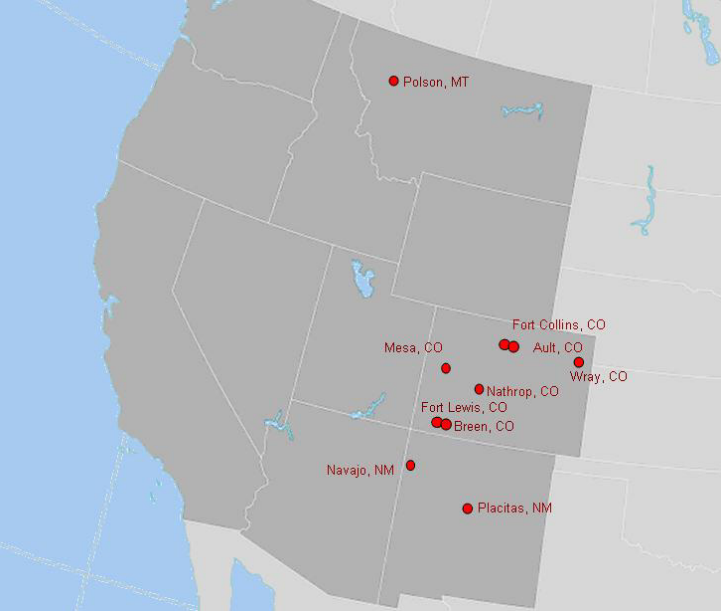
**Map of western United States showing locations of trapping sites at which rodents with Sin Nombre virus RNA were obtained**.

**Figure 2:**
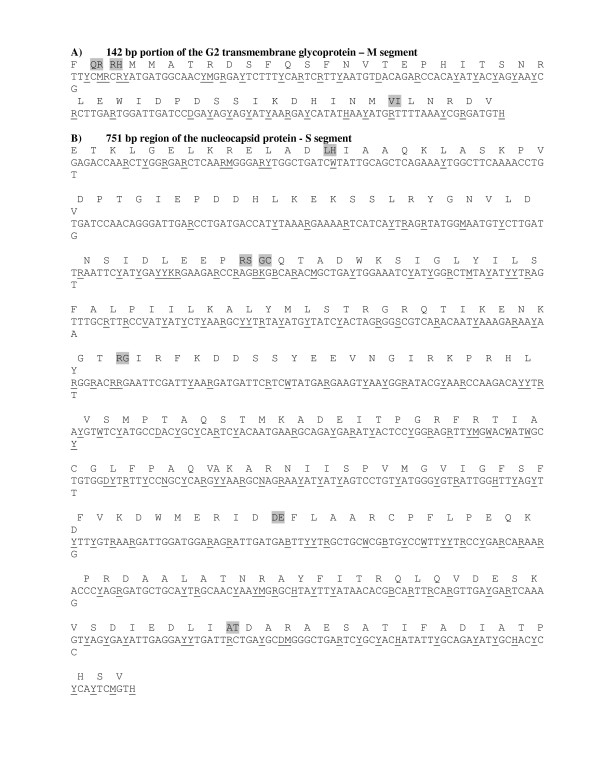
**Consensus sequences for the 142 nt portion of the G2 transmembrane glycoprotein and the 751 nt region of the nucleocapsid protein.** Polymorphic sites are underlined. The predicted amino acid sequence appears above each codon. Amino acid replacements are highlighted in gray.

We sequenced portions of both the S and M segments of SNV RNA samples collected from deer mice at six locations in Colorado, two locations in New Mexico and one location in Montana (Table 1 and Figure 1). PCR products were obtained for both M and S segments of SNV RNAs of 20 peromyscine rodents. These were then cloned and sequenced; a minimum of three clones per sample were sequenced to derive a consensus. Consensus sequences for the 142 nt portion of the G2 transmembrane glycoprotein and the 751 nt region of the nucleocapsid protein are shown in Figure 2. Polymorphic sites are underlined. The predicted amino acid sequence appears above each codon. Replacement substitutions are highlighted in gray.

**Table 1:** Mouse species, identification and accession numbers, date and the city nearest to the trapping site

Species	ID	Acc. no.	Capture Date	Location	S	M
*Peromyscus maniculatus*	M02	MN-2	07/21/2003	Mesa, CO	+^a^	+
*Peromyscus maniculatus*	M06	BBE-13	06/09/2004	Breen, CO	+	+
*Peromyscus maniculatus*	M11	B-942	07/05/2003	Polson, MT	+	+
*Peromyscus maniculatus*	M12	NK-62732	02/07/1995	Placitas, NM	+	+
*Peromyscus boylii*	M15	NK-86435	05/21/1999	Navajo, NM	+	+
*Peromyscus maniculatus*	M16	NK-86747	07/14/1999	Navajo, NM	+	+
*Peromyscus maniculatus*	M17	NK-97143	12/05/2000	Navajo, NM	+	+
*Peromyscus maniculatus*	M19	FC-8	04/04/2006	Fort Collins, CO	+	+
*Peromyscus maniculatus*	M20	ES-7	07/11/2006	Ault, CO	+	-
*Peromyscus maniculatus*	M22	TS-830-18	08/30/2006	Fort Lewis, CO	+	+
*Peromyscus maniculatus*	M23	TS-830-20	08/30/2006	Fort Lewis, CO	+	+
*Peromyscus maniculatus*	M24	TS-830-08	08/30/2006	Fort Lewis, CO	+	+
*Peromyscus maniculatus*	M25	TS-830-09	08/30/2006	Fort Lewis, CO	+	+
*Peromyscus maniculatus*	M27	TS-830-06	08/30/2006	Fort Lewis, CO	+	+
*Peromyscus maniculatus*	M28	C-1	09/13/2006	Nathrop, CO	+	+
*Peromyscus maniculatus*	M29	C-8	09/13/2006	Nathrop, CO	+	+
*Peromyscus maniculatus*	M30	J-9	09/13/2006	Nathrop, CO	+	+
*Peromyscus maniculatus*	M31	J-23	09/13/2006	Nathrop, CO	+	+
*Peromyscus maniculatus*	M32	2C-4	09/14/2006	Nathrop, CO	+	+
*Peromyscus maniculatus*	M33	WR-7	06/05/2007	Wray, CO	+	+
*Peromyscus maniculatus*	M34	WR-11	06/05/2007	Wray, CO	+	+
*Peromyscus maniculatus*	M37	WR-20	06/05/2007	Wray, CO	-	+
**TOTAL**					22	22

^a ^"+" indicates mice from which S or M RNAs were successfully sequenced

### Phylogenetic analysis

**Figure 3:**
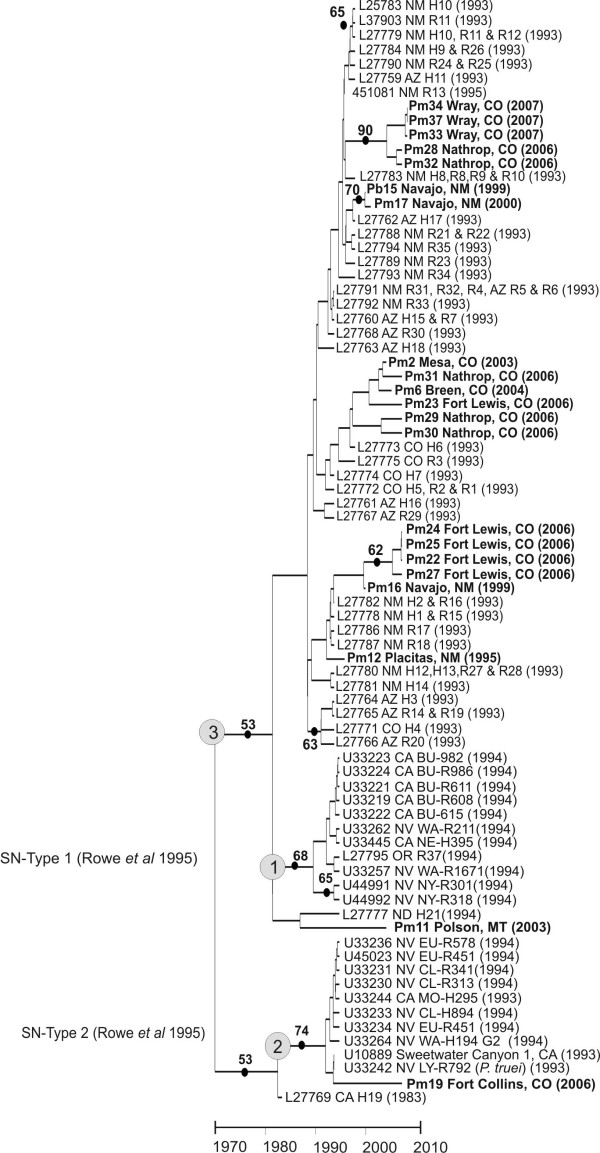
**Maximum likelihood tree for the M segment with 1,000 bootstrap pseudoreplications**. New sequences from the present study are in bold. The state and date of collection are listed for each sequence. All clades with bootstrap support > 50% are indicated with a dot and the % of bootstrap support. The SN-Type clades 1 and 2 proposed by Rowe *et al*. (1995) are indicated with grey circles as is clade 3 in which all but one of the sequences in the present study arose.

The 142 nt amplicon region of the M segment encoded a 47 codon portion of the G2 transmembrane glycoprotein and was sampled from 21 mice. An additional 55 sequences of the same region of the M segment were added from GenBank to provide a geographic and temporal context for our sequences. Table 2 lists the model and parameters estimated in Modeltest 3.7 used to derive the phylogeny shown in Figure 3. This is the rooted, Maximum Likelihood (ML), time-based phylogeny inferred using a strict molecular clock in BEAST 1.4 [11] for the M segment. There were 37 parsimony informative sites in the M segment and consequently the bootstrap support for the various clades was low. The two clades labeled with light grey circles correspond to the SNV-type clades 1 and 2 proposed by Rowe *et al*. [12] from SNV collections from Nevada and California. Pm11 from Montana is basal to SNV-type Clade 1, whereas Pm19 is basal to SNV-type Clade 2. However, the remainder of our sequences arose on the clade labeled 3, as did most of the published sequences that have been collected from Arizona and New Mexico.

**Table 2:** Rate and shape parameters estimated by Modeltest 3.7 for each of the four phylogenies presented in Figures. 3, 4, and 8

	Transition/transversion ratio		Kappa		Model		Shape parameter (α)			
Phylogeny										
Figure 2 M segment	-		-		GTR + Γ		0.1401			
Figure 3 S segment	-		-		GTR + Γ		0.1675			
										
Figure 8 M segment	7.7887		15.58		K80(K2P) + Γ		0.0004			
Figure 8 S segment	-		-		GTR + Γ		0.1693			
										
	Substitution rate matrix						Proportion of each nucleotide			

Phylogeny	AC	AG	AT	CG	CT	GT	A	C	G	T

Figure 2 M segment	1.000	9.889	0.176	0.176	9.889	1.000	0.339	0.181	0.167	0.314
Figure 3 S segment	2.349	18.668	1.041	0.258	26.663	1.000	0.302	0.196	0.224	0.278
										
Figure 8 M segment	-	-	-	-	-	-	0.250	0.250	0.250	0.250
Figure 8 S segment	1.000	11.321	0.522	0.522	18.495	1.000	0.306	0.194	0.221	0.279

**Figure 4:**
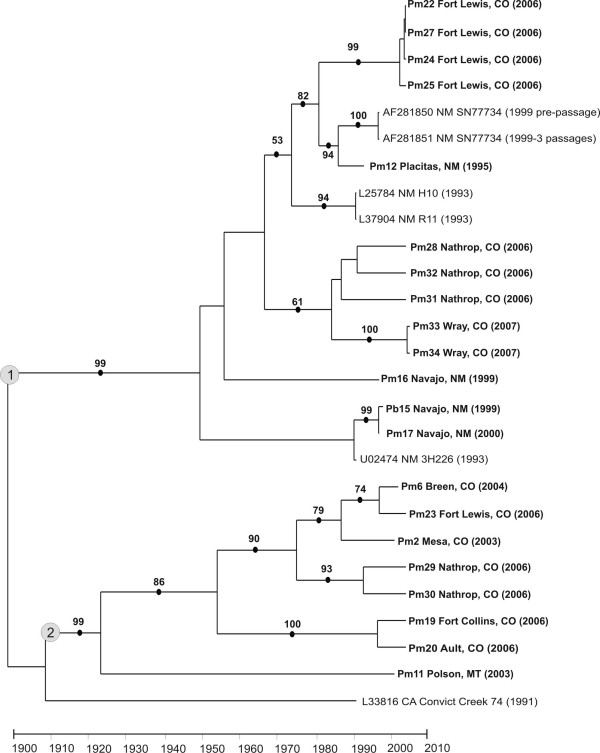
**Maximum likelihood tree for the S segment with 1,000 bootstrap pseudoreplications**. New sequences from the present study are in bold. The state and date of collection are listed for each sequence. All clades with bootstrap support > 50% are indicated with a dot and the % of bootstrap support. Clades 1 and 2 referred to in the text are indicated with grey circles.

The 751 nt region of the S segment encoded 250 codons of the highly conserved nucleocapsid protein and was sampled from 21 mice. This region of the S segment has not been as widely used as has the M segment in prior studies so that only six additional sequences from the literature were available. Table 2 lists the model and parameters estimated in Modeltest 3.7 used to derive the phylogeny shown in Figure 4. This is the rooted, ML, time-based phylogeny inferred using a strict molecular clock with BEAST 1.4 for the S segment. There were 211 parsimony informative sites, and bootstrap support for the various clades was high. The two clades indicated with light grey circles are well supported. Clade 1 contains 13 of our S segment sequences and all previously published S segment sequences from New Mexico. The "Four Corners hantavirus" (Sin Nombre virus) sequence reported by Hjelle et al. 1994 [13] is basal to Clade 1. Clade 2 is a new clade containing exclusively Colorado sequences. Interestingly, basal to Clade 2 is the SNV sequence from a deer mouse captured at Convict Creek, California [14].

Clades from both segments were examined with respect to geographic origin of the samples. Viruses from at least two different clades were co-circulating in Fort Lewis deer mice and the same was true in deer mice from Nathrop, CO and Navajo NM.

### Rates and patterns of molecular evolution

The M segment dataset was analyzed with all 78 sequences shown in Figure 3 (Table 1). The absolute rate of molecular evolution of the M segment was 6.76 × 10^-3 ^substitutions/site/year. The absolute age of the M segment tree was estimated to be 37 years; a time scale in years appears at the bottom of Figure 3. In the S segment, the rate of molecular evolution was 1.93 × 10^-3 ^substitutions/site/year and the absolute age of the tree was 106 years (Figure 4). The substitution rates (π, π_s_, π_a_) in the two segments were similar (Table 3). The estimated ages of either segment suggest that SNV arose recently, within the past 37–106 years.

**Table 3:** Polymorphism and substitution rates in the M and S sequences of SNV utilized in Figures 3 and 4

Segment analyzed	No. of sequences (this study)	No. of unique sequences	Haplotype diversity ± std. dev
M segment	78 (21)	65	0.993 ± 0.004
S segment	27(21)	22	0.977 ± 0.019
			

Segment analyzed	π ± std. dev	π_s_ (potential synonymous sites)	π_a_ (potential replacement sites)

M segment	0.07525 ± 0.00365	0.378 (27.5)	0.00070 (110.6)
S segment	0.07432 ± 0.00693	0.312 (176)	0.00173 (574)

### Linkage disequilibrium

**Figure 5:**
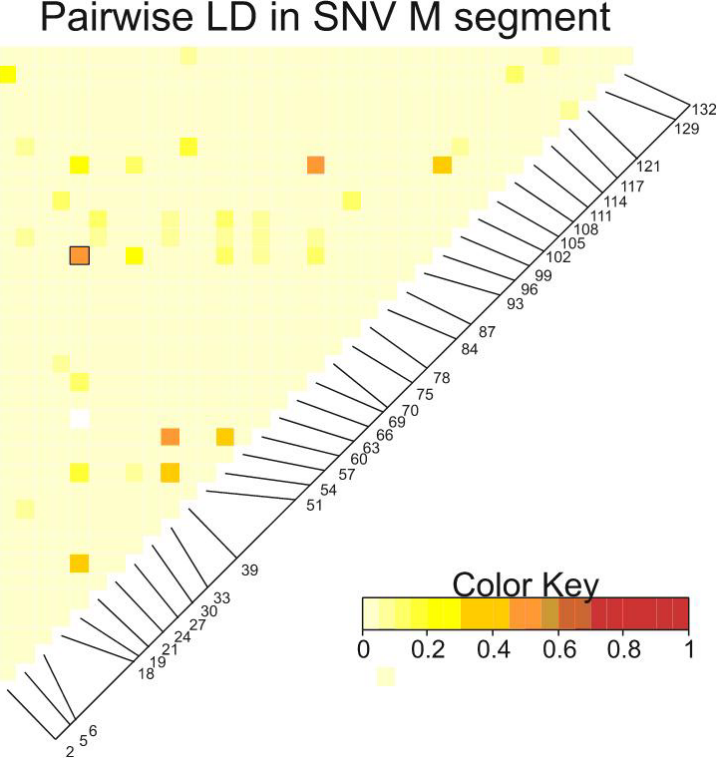
**A heat map of linkage disequilibrium among the 36 polymorphic sites in the M segment**. Only sequences from clade 3 (Figure 3) were analyzed. The matrix is read according to the nucleotide position of segregating sites displayed along the diagonal. Small disequilibrium coefficients are represented by white or yellow and large disequilibrium coefficients are represented by orange or red.

Figure 5 is a heat diagram in which small disequilibrium coefficients are represented by white or yellow and large disequilibrium coefficients are represented by orange or red. The matrix is read according to the nucleotide position of segregating sites displayed along the diagonal. For example in Figure 5, the square connecting sites 19 and 96 is orange (and placed in a box); this corresponds to an r^2 ^of 0.596 and these sites are in significant linkage disequilibrium. The boxes linking sites 33 with 60 or 69 with 111 are also orange indicating that these sites are also in disequilibrium with one another. In contrast, squares linking site 2 with all other sites are white or light yellow and these sites are in equilibrium with site 2. The majority of boxes in Figure 5 are light, suggesting that most segregating sites in the M segment exist in equilibrium. This probably occurs because mutations at segregating sites in this region of the M segment occur independently of one another.

**Figure 6:**
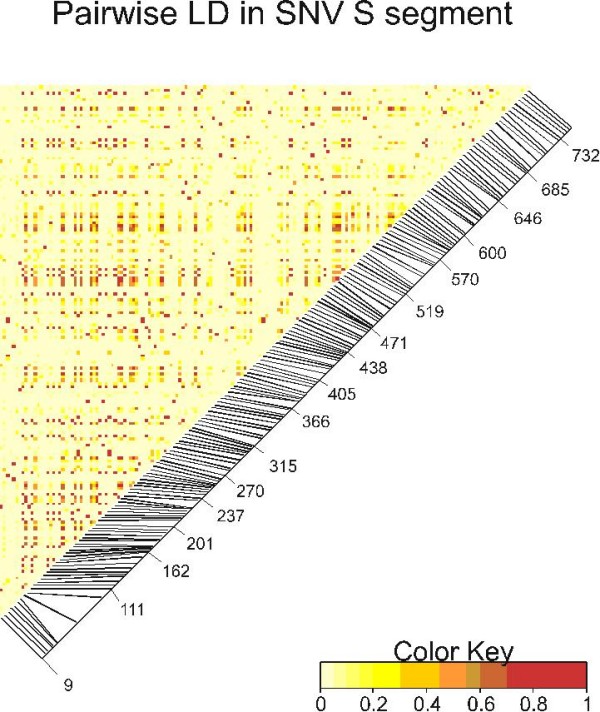
**A heat map of linkage disequilibrium among the 177 polymorphic sites in the S segment**. Only sequences from clades 1 and 2 (Figure 4) were analyzed. The matrix is read according to the nucleotide position of segregating sites displayed along the diagonal. Small disequilibrium coefficients are represented by white or yellow and large disequilibrium coefficients are represented by orange or red.

Analysis of the S segment indicates many orange and red boxes suggesting a high rate of disequilibrium distributed throughout the S segment sequence (Figure 6). These patterns suggest that our sampling of only a 142 nt portion of the M segment may not provide an accurate sample of evolutionary rates and patterns in the whole M segment. Many sites in the S segment are in disequilibrium and our coverage of this segment thus appears adequate.

### Test of neutrality

**Figure 7:**
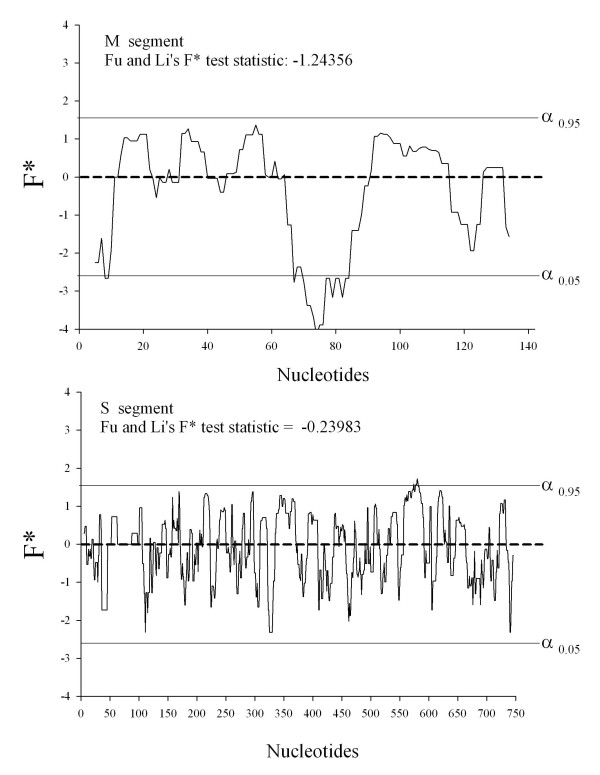
**F test of neutrality (Fu and Li, 1993) for the M and S segments**. Only sequences from clade 3 of the M tree (Figure 3) and clades 1 – 2 of the S tree (Figure 4) were analyzed.

The uniformly high synonymous substitution rate (π_s_) in the M and S segments shown in Table 3 suggests a very high nucleotide substitution rate but a very low rate of amino acid substitutions. This pattern is consistent with strong purifying selection. To test this pattern further, the F* statistic [15] was calculated to test for selective neutrality. Figure 7 shows that the overall F* statistic for the M segment is negative and that the regions that are significant in the 3' region are negative as well. The overall F* statistic for the S segment is a smaller negative number but only a small region is significant. Recalling that F* > 0 under balancing selection, F* ≈ 0 with neutral substitutions and F* < 0 under purifying selection, Figure 7 supports a model of purifying selection for the M segment and neutral substitutions in the S segment.

### Segment reassortments

**Figure 8:**
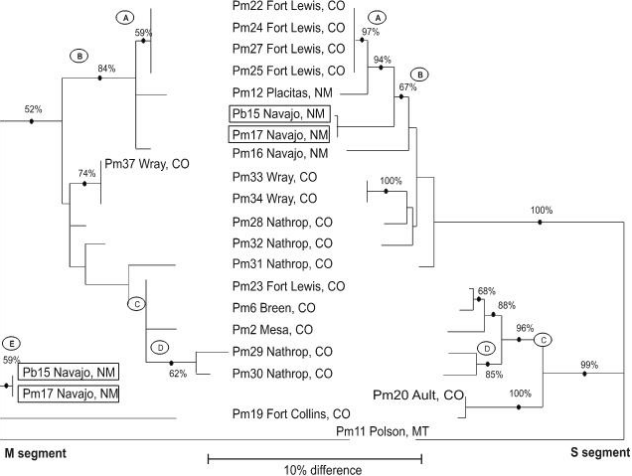
**Maximum likelihood trees for the M and S sequences collected in this study from the same mice**. Bootstrap results are from 1,000 pseudoreplications. The names of samples that arose from the same mouse and appear in the same clade are listed once in the center of the diagram. The names of samples that arose from the same mouse but appear in different clades are shown in boxes.

Maximum likelihood trees were created for both genome segments (M segment on left, S segment on right in Figure 8). Specific clades in the M and S segment trees are labeled by letters in ovals from A-E, and A-D, respectively. For reasons already discussed, the majority of bootstrap values in the M segment phylogeny were low, whereas the bootstrap scores in the S segment phylogeny are large.

There are two isolates in which the M segment arises on a different branch than does the S segment. Pb15 and Pm17 both from Navajo, NM, arose from Clade E in the M segment but arose from Clade B in the S segment. Thus, 2 of the 20 peromyscines from which we amplified both the S and M segments appeared to contain reassortant viruses. Otherwise, the S and M segment phylogenies appear to parallel one another. A χ^2 ^test of independence was performed to examine the overall correlation between the M and S segment sequences from same individuals. The χ^2 ^test was highly significant (P ≤ .0001) as was the Fisher's Exact Test (P = 7.96 × 10^-9^). This suggests that the M and S segment sequences from the same mice tended to arise on the same clade.

## Discussion

Phylogenetic analyses of SNV genotypes revealed that 10% (2/20) of viruses were reassortants, not significantly less (Fisher's Exact Test P = 1.00) than the 14% (6/46) reported previously [9] in SNV sequences from Nevada and eastern California. Those authors examined isolates from 3 humans and from 43 rodents and found that all of the human isolates but only three of the rodent isolates were reassortants. A better comparison therefore is 3 of 43 (7%) but this also is not significantly less than the rate in the present study (Fisher's Exact Test P = 0.6488).

Henderson et al. [9] suggested that as genetic distance increases, the frequency of formation of viable reassortants decreases and that hantaviruses which are primarily maintained in different rodent hosts rarely have the opportunity to genetically interact. Our data only partially support this suggestion. Notice for example in Figure 8 that the M segment of Pb15 (from a brush mouse) and Pm17 from Navajo, NM (Clade F) are genetically distant (4% difference) from M segments of those in Clade B, the clade containing the S segment of Pb15 and Pm17. Acquisition of SNV by a brush mouse likely was due to a spill-over event, an infrequent interspecies interaction between this rodent and an SNV-infected deer mouse. Alternatively, it may be that rodents in species-poor areas are spared frequent contact with rodents in nearby but not contiguous areas. Further interpretations require additional information regarding climatic conditions, habitat peculiarities and physical barriers, and information about seasonality of collections.

Very few sites in the 142 nt of the M segment were in linkage disequilibrium (Figure 5) while many of the sites within 150 nt of one another in the S segment were in disequilibrium (Figure 6). The differences in disequilibrium rates are not attributable to greater mutation rates because both segments have similar evolutionary rates (Table 3). The differences could be related to relative synonymous codon usage; the S segment having a biased and therefore constrained usage pattern, while the M segment may have had unbiased usage. However, a scaled χ^2 ^analysis of relative synonymous codon usage in DNAsp revealed no bias in either gene (analysis not shown). The difference might be associated with the relative ages of the two sequences since the S segment was estimated to be 2.86 times (106 years/37 years) older than the M segment. As an ancestral sequence accumulates mutations, distinct lineages begin to form. Initially sequences may be in disequilibrium because segregating sites have not had sufficient time to accumulate reverse mutations. However, given enough time, these reverse mutations will accumulate and patterns of disequilibrium will dissipate. However this is opposite to the observed trend; S is older than M. There may be some type of epistatic selection acting across the nucleocapsid gene or protein that maintains polymorphic sites in disequilibrium while no such selection is acting upon the G2 gene or glycoprotein. However, we have no hypotheses about the form of such a selection.

The significance of these findings lies in the observations regarding the relatively high rate of reassortment. The deer mouse is the most common and most numerous mammal in North America. It occurs throughout the United States and much of Canada, except for their eastern coasts. Because SNV is transmitted principally through transfer of saliva, urine or feces from SNV-infected rodents, because these rodents are so numerous, and because the virus affects the rodent host but does not do so critically [16], intraspecies transmission of SNV occurs at high frequency [17,18]. This provides frequent opportunities for genomic evolution to occur via reassortment, as has been reported for influenza viruses [19].

If one arbitrarily selects a location in North America and sequences the M and S RNAs of SNV from deer mice at that site and then sequences M and S RNAs from deer mice at sites increasingly distant (geographically or by habitat type) from that site, numerous and divergent genotypes likely would be found. Indeed, the initial epidemiologic studies of SNV (S.T. Nichol, personal communication, 1994) showed such a pattern on a smaller geographic scale. The number of mutations and cumulative reassortments mount until, at the greatest geographic distances, the virus might be seen as being no longer consistent with the topotype. Host-switching events may lead to distinct variants in different peromyscine subspecies (c.f., Monongahela virus in *P. maniculatus nubiterrae*) or in rodents of different peromyscine species (c.f., New York and Blue River viruses in *P. leucopus*). The phylogeography of these subtypes and varieties must be determined, if we are to understand rodent host and hantaviral genetics because virus variations may reflect those of their rodent hosts, as has been suggested by Dragoo et al. [4].

It appears to be counterintuitive that this virus has evolved as rapidly as our data suggest. One might justifiably ask how this virus has managed to become distributed so widely in North America only recently, when its host rodent, the deer mouse, is and has been distributed over this continent for a very long time. Could a progenitor of SNV have been a virus whose rodent host was not the deer mouse and which switched hosts only fairly recently? Low rates of nucleotide substitutions have been hypothesized for the hantaviruses but, as Ramsden et al. have suggested, "hantaviruses replicate with an RNA-dependent RNA polymerase, with error rates in the region of one mutation per genome replication, [and therefore] this low rate of nucleotide substitution is anomalous" [20]. Do only slight host genetic differences lead to only slight, but significant, differences in the virus? Can such apparently trivial virus genetic differences have substantial epidemiologic differences, perhaps effecting pathogenicity? There are many possible scenarios that should be investigated; the data we present here do not shed light on them.

Variants that are widely divergent may have acquired a gene or genes, one or more mutations, or combinations of otherwise non-pathogenic changes, and changes thereby arise and may have epidemiologic consequences. Such changes could be towards or away from pathogenicity, infectivity, stability, persistence, host adaptability, replication, or otherwise. These combinations of events are random, or at least not predictable at this time, and therefore continued surveillance is needed.

## Methods

### Rodent sampling

Using Colorado State University Animal Care and Use Committee-approved procedures, rodents of several species were captured using Sherman live-traps. Trapping was conducted at several geographically diverse locations in Colorado, including Fort Collins and Ault (north-central), Wray (northeast), Fort Lewis and Breen (southwest), Nathrop (central), and Mesa (west-central) (Figure 1). Habitats at the Fort Collins and Wray sites are characterized as shortgrass prairie; at Fort Lewis and Breen as montane shrubland dominated by Gambel's oak (*Quercus gambelii*) and big sage (*Artemisia tridentata*); at Mesa and Nathrop as pinyon-juniper (*Pinus edulis *and *Juniperus *spp.) and sagebrush shrublands; the Ault site was an uncultivated agricultural field.

One SNV-infected deer mouse from Polson, Montana was kindly provided by Dr. Richard Douglas, Montana Tech, Butte, Montana. Several others were from Navajo and Placitas, New Mexico, gifts of Dr. Terry Yates, University of New Mexico, Albuquerque. Deer mice trapped in Colorado were sacrificed and liver, lung, kidneys and spleen were removed and stored in RNALater (Ambion, Austin, TX) at -70°C until they were analyzed.

### Collecting and processing deer mice

Deer mice were captured in 8 × 9 × 23-cm non-folding Sherman live-traps (H. B. Sherman Traps, Inc., Tallahassee, FL) baited with cracked corn, peanut butter and rolled oats. Animals were anesthetized with isoflurane, standard measurements were taken and blood samples were collected from the retro-orbital plexus. For animals captured in Colorado during and after 2006, a 1-hour rapid ELISA was used in the field to test deer mouse blood samples for antibody to SNV [21]. Seropositive deer mice were euthanized and kidney, liver, lung, and spleen samples were collected from each. Those found to be seronegative were released at the site of capture. Prior to 2006, only carcasses of deer mice that had died under anesthesia and were later found to be seropositive by an ELISA were used in this study.

### RNA purification and reverse transcription

For total RNA extraction, tissues were frozen in liquid nitrogen and then homogenized using a mortar and pestle. Homogenates were extracted once using guanidinium thiocyanate-phenol-choloroform (Trizol, Invitrogen, Carlsbad, CA); RNA was precipitated with isopropanol. Total RNA from infected mouse tissue was then reverse transcribed with Thermoscript (Invitrogen) and amplified via polymerase chain reactions (PCR) using segment specific primers (Table 4). Amplicons of a 751 nt region of the S segment and of a 142 nt region of the M segment were produced. Nested PCR was then performed to produce samples used as sequencing templates.

**Table 4:** Primers used in reverse transcription and amplification of portions of the S and M segments of Sin Nombre virus genotypes

PCR step	SNV segment	Primer name	Sequence
RT	S	SNV-S5'T	TAGTAGTAGACTTCKTRAAGAGCTACT
1° reaction	S	SNV-S41s	GGAATGAGCACCCTCAAAGAAGTGCAAGACAAC
1° reaction	S	PSE-S1064r	ATRGTRTTYCTCATATCCTG
2° reaction	S	SNV-S143s	TGGACCCMGATGAYGTTAACAA
2° reaction	S	SNV-S1000r	GACAYCGATCWGGNGCACATGCAAARACCC
RT	M	SNV-M2730s	CTTTTAGAAAGAWMTGTGSRTTTGC
1° reaction	M	SNV-M2730s	CTTTTAGAAAGAWMTGTGSRTTTGC
1° reaction	M	SNV-M3023r	CCTACTCCTGAACCCCAGGCCCCG
2° reaction	M	SNV-M2764s	CCAACATGTGAGTATCAAGGCAACACAGTGCTGG
2° reaction	M	SNV-M2966r	GGKKTWTCACTTAGRTCYTGRAAGG

The S segment was amplified with the following cycling parameters: 94°C (120 s) initial denaturing, [94°C (30 s), 58°C (30 s), 72°C (60 s)] 40 times, and a final extension at 72°C (300 s). The M segment was amplified with these cycling parameters: 94°C (120 s) initial denaturing, [94°C (10 s), 56°C (15 s), 72°C (30 s)] 40 times, and a final extension at 72°C (300 s). Table 4 shows the primers used for each segment. Purified PCR products were cloned into vector pCR2.1 (TOPO cloning kit, Invitrogen). A minimum of 3 clones were sequenced per sample to control for any Taq polymerase-induced mutations. Samples were fully sequenced by the Colorado State University Macromolecular Sequencing core facility. Cloned segment data were submitted for BLAST analysis to confirm their identities as SNV sequences .

### Phylogenetic Analysis

To compare our sequences with those reported in the literature we used the following M segment sequences: L27759-27795 [22], U33219-U33264, U45023 [12], U10889 [23], L37903 [24], and U44991 – U44992 from [9]. S segment sequences were U47135 [25], U29210 [26], U09488 [27], AF281850 and AF281851 [28], L33683 and L33816 [14], L37904 [23], U02474 [13] and L25784 [21]. Maximum likelihood trees were estimated separately for the M and the S segments by first identifying the evolutionary model that best fits the data, using Modeltest 3.7 [29] with the Phylogenetic Analysis Using Parsimony (PAUP) 4.0b10 package [30]. The optimal model and parameters were then used to estimate the ML tree in PAUP. Bootstrap values of individual branches were obtained with ML analysis of 100 pseudoreplicates. This same model and parameters were then used in the BEAUTi/BEAST 1.4 package to develop a rooted, time-based phylogeny inferred using a strict molecular clock. The year of collection minus 2007 values were entered as "Date before the present" variables for each RT-PCR or isolation record. TreeAnnotater (v1.4.8) was used to generate the ML tree. The rate of molecular evolution (substitutions/site/year) was also estimated from the BEAUTi/BEAST 1.4.

### Linkage disequilibrium analysis

Linkage disequilibrium is a measure of the degree to which substitutions in a segment occur independently of one another. Substitutions that occur together in a segment at a rate predicted by their independent frequencies are in linkage equilibrium. Substitutions that occur more or less often than expected by random chance are considered to be in linkage disequilibrium. The Hill and Robertson correlation coefficient (r^2^) [31] was used as a metric of disequilibrium because it ranges from zero (linkage equilibrium) to one (linkage disequilibrium). Linkage disequilibrium also tests whether sampling a portion of a genome segment is representative of the whole segment. Linkage equilibrium is detected when different parts of a segment are evolving independently and sequencing a portion of the segment may not provide a representative sample of the whole. Linkage disequilibrium patterns among all polymorphic sites were plotted on a heatmap using the LDheatmap program in R [32].

### Nucleotide polymorphisms and substitution rates

For each segment, the computer program DnaSP 4.5 [33] estimated haplotype diversity [34] (equations 8.4 and 8.12 but replacing 2n by n) and π the average number of nucleotide differences among all pairwise comparisons of sequences [34] (equation 10.5). π also was estimated separately for synonymous (π_s_)and replacement substitutions (π_a_).

A test of selective neutrality was performed [15]. The F* statistic was calculated in DnaSP to provide a normalized comparison of the number of all mutations (η) to the number of singletons (η_s_). This analysis assumes that F* > 0 (η > η_s_) under balancing selection, F* ≈ 0 (η ≈ η_s_) with neutral substitutions and F* < 0 (η < η_s_) under purifying selection.

### Segment reassortants

The topology of the M and S segment trees were compared to detect whether SNV RNA sequences from the same mouse arose in the same branches. Sequences arising in different clades were considered *prima facie *evidence of reassortment. A χ^2 ^test of independence was also performed to test whether M and S segments from the same mouse were significantly correlated (non-independent). This was done by labeling clades with a letter and then assigning each isolate the letter of its clade in the M and S segment trees.

## Competing interests

The authors declare that they have no competing interests.

## Authors' contributions

WCB performed the statistical analyses and wrote descriptions of the techniques and results. JBD assisted in conducting the field studies and conducted the molecular genetics studies and the sequence alignments. MTH supervised the molecular analyses and sequence alignments and participated in the field studies. BJB conceived the study and provided advice regarding additional field work. CHC supervised and participated in the field studies and drafted the manuscript. All authors read and approved the final manuscript.
